# Stress‐induced changes in abundance differ among obligate and facultative endosymbionts of the soybean aphid

**DOI:** 10.1002/ece3.1908

**Published:** 2016-01-18

**Authors:** Laramy S. Enders, Nicholas J. Miller

**Affiliations:** ^1^ Department of EntomologyUniversity of Nebraska‐Lincoln103 Entomology HallLincolnNebraska68583‐0816

**Keywords:** *Arsenophonus*, *Buchnera*, plant defense, quantitative PCR, *Wolbachia*

## Abstract

Bacterial endosymbionts can drive evolutionary novelty by conferring adaptive benefits under adverse environmental conditions. Among aphid species there is growing evidence that symbionts influence tolerance to various forms of stress. However, the extent to which stress inflicted on the aphid host has cascading effects on symbiont community dynamics remains poorly understood. Here we simultaneously quantified the effect of host‐plant induced and xenobiotic stress on soybean aphid (*Aphis glycines*) fitness and relative abundance of its three bacterial symbionts. Exposure to soybean defensive stress (*Rag1* gene) and a neurotoxic insecticide (thiamethoxam) substantially reduced aphid composite fitness (survival × reproduction) by 74 ± 10% and 92 ± 2%, respectively, which in turn induced distinctive changes in the endosymbiont microbiota. When challenged by host‐plant defenses a 1.4‐fold reduction in abundance of the obligate symbiont *Buchnera* was observed across four aphid clonal lines. Among facultative symbionts of *Rag1*‐stressed aphids, *Wolbachia* abundance increased twofold and *Arsenophonus* decreased 1.5‐fold. A similar pattern was observed under xenobiotic stress, with *Buchnera* and *Arsenophonus* titers decreasing (1.3‐fold) and *Wolbachia* increasing (1.5‐fold). Furthermore, variation in aphid virulence to *Rag1* was positively correlated with changes in *Arsenophonus* titers, but not *Wolbachia* or *Buchnera*. A single *Arsenophonus* multi‐locus genotype was found among aphid clonal lines, indicating strain diversity is not primarily responsible for correlated host‐symbiont stress levels. Overall, our results demonstrate the nature of aphid symbioses can significantly affect the outcome of interactions under stress and suggests general changes in the microbiome can occur across multiple stress types.

## Introduction

There is a growing appreciation of the complex evolutionary networks, involving a spectrum of mutualistic, conditionally beneficial and parasitic interactions between insects and microbes (Frago et al. 2012; Sugio et al. 2014). Bacterial symbionts can manipulate host reproduction (Duron et al. 2008), promote adaptive divergence (Janson et al. 2008; Hansen and Moran 2014) and mediate host response to various forms of environmental stress (Oliver et al. 2010). The insect microbiome therefore represents a reservoir of novel traits, with the potential to influence the ecological and evolutionary dynamics of natural populations.

Investigating insect‐microbial interactions under environmental stress can begin to illuminate the role symbionts play in adaptive processes. Unraveling the molecular basis of mutualistic and defensive symbioses has received considerable attention (Dunbar et al. 2007; Oliver et al. 2009; Hansen and Moran 2014), however eco‐evolutionary aspects are relatively unexplored (Kwiatkowski and Vorburger 2012; Russell et al. 2013; Oliver et al. 2014). Currently, we lack an understanding of how selective pressure resulting from external environmental stress alters symbiont community structure or contributes to host‐symbiont coevolution. Stress can be functionally defined as any factor causing a reduction in organismal fitness relative to benign conditions, which enables broad scale quantification of the impacts of stress (Fox and Reed 2011; Enders et al. 2015; Schulte 2014). We propose the adverse effects of stress can be measured as changes in both insect fitness components and endosymbiont abundance, which reflect host physiological impairment and either increased death rates or reduced growth rates in bacterial populations. Within this multi‐level framework, the nature of the symbiotic relationship is likely an important determinant of microbial response to changes in the internal host environment resulting from stress exposure (Pan et al. 2013; Martinez et al. 2014). Stress‐induced reductions in abundance may be greatest for obligate symbionts in tight association with the host, such as those required for basic metabolic functions. In contrast, parasitic symbionts may increase in abundance under stress if the host immune system is weakened (Berticat et al. 2002; Pan et al. 2013). Studies simultaneously examining insect and microbial stress responses thus provide a system‐wide approach to uncovering the basis of symbiont contributions to host phenotypic diversity.

Aphids harbor a diverse bacterial community that includes both obligate and facultative associations. The obligate nutritional symbiont *Buchnera aphidicola* is required for survival and shares a long evolutionary history with the aphid host (Moran et al. 1993; Hansen and Moran 2011). A multitude of facultative symbionts have also been shown to provide adaptive benefits under stressful conditions, such as enhanced thermotolerance and protection against pathogens and parasites (see review by Oliver et al. 2010). In addition, evidence continues to mount demonstrating the outcome of aphid‐host plant interactions critically depends on microbial associations (Tsuchida et al. 2004; Ferrari et al. 2007; Francis et al. 2010). A prevailing source of biotic stress for aphids arises from the intimate and often antagonistic relationship with the host plant. Plants possess morphological and chemical defenses that impose considerable stress on herbivores by decreasing survival and inhibiting growth and reproduction (Chen 2008; Howe and Jander 2008; Smith et al. 2009). Damage inflicted by plant defenses may also produce cascading effects on the interactions between insects and their microbes, thus affecting symbiont abundance and community structure (Biere and Bennett 2013). Similarly, exposure to insecticidal chemicals commonly used to control aphid populations in agro‐ecosystems could trigger changes in the microbiome as a by‐product of physiological stress imposed on the aphid host. Toxins that target critical functions, such as transmission of nerve impulses, cellular respiration and lipid biosynthesis, cause broad scale impairment and cellular damage (Foster et al. 2007; Simon 2011) that are likely to adversely affect both host and symbiont community. Aphids also lack known antimicrobial peptides that control endosymbionts in other insects (Gerardo et al. 2010), which may enable some endosymbionts to increase in abundance under stressful conditions.

Although the microbiome of the pea aphid (*Acyrthosiphon pisum)* is well characterized, the functional significance of symbiotic relationships in most aphid species remains poorly understood. For example, the soybean aphid (*Aphis glycines*) harbors *Buchnera* and two facultative endosymbionts, *Arsenophonus* and *Wolbachia* (Liu et al. 2012; Bansal et al. 2014a) that are rare or have not been detected in pea aphid populations. *Buchnera* provides essential amino acids lacking from plant phloem, but the role of facultative symbionts in soybean aphid biology is unknown. *Aphis glycines* is a cyclically parthenogenetic species native to east Asia that specializes on soybean (*Glycine max*) and has become a major agricultural pest in North America since being introduced around 2000 (Hill et al. 2012). In native and invasive soybean aphid populations the frequency of *Arsenophonus* is widespread (Wulff et al. 2013; Bansal et al. 2014a), but does not appear to confer resistance to parasitoids or fungal pathogens based on research comparing artificially cured versus infected individuals (Wulff et al. 2013). Recent work also found a general fitness benefit associated with *Arsenophonus* infection, but no evidence this symbiont is the primary factor driving phenotypic differences in aphid virulence to soybean plant defenses (Wulff and White 2015). In contrast, *Wolbachia* frequencies in natural populations have not been investigated and it is unclear what role this symbiont might play in soybean aphid biology. It is also unknown whether either *Arsenophonus* or *Wolbachia* mediate other stress responses, for example, involving exposure to toxins. In addition, considerable effort has been made to determine whether aphid stress response is altered in the presence of specific symbionts, while less attention has been given to understanding how the microbiome responds to stress. It remains unclear whether changes in symbiont population dynamics are unique to different stressors or associated with phenotypic variation in aphid stress tolerance.

This study investigates the effect of host‐plant defenses and xenobiotic stress on the soybean aphid and its bacterial endosymbiont community. Specifically we addressed the following questions: (1) Do obligate and facultative symbionts respond differently to stress imposed on the aphid host? (2) Do different stress types produce unique changes in the microbiome? and (3) Are the effects of stress correlated in aphids and their bacterial symbionts? We employed a multi‐level approach that measured the effect of plant defensive stress inflicted through expression of the soybean *Rag1* (*Resistance to Aphis glycines*) gene and exposure to a neurotoxic insecticide (thiamethoxam) on aphid fitness and endosymbiont relative abundance. Quantitative PCR (qPCR) was used to estimate changes in the titer of the three *A. glycines* bacterial symbionts (*Arsenophonus*,* Buchnera*, and *Wolbachia*). In addition, we exploited natural aphid clonal variation in stress tolerance to determine whether the effects of stress are correlated between host and symbionts.

## Methods and Materials

### Aphid rearing and maintenance

Experiments were conducted using four parthenogenetic clonal lines that were each founded from a single apterous female. Lines 1 and 2 were isolated in 2013 from a laboratory colony obtained from the National Soybean Research Laboratory, University of Illinois, Urbana‐Champaign. Lines 3 and 4 were isolated from a soybean field collection in Wisconsin in 2011 (Enders et al. 2014). All lines were continuously maintained on a single soybean plant (variety KS4202) grown in plastic Cone‐tainers (Ray Leach Conetainer; Hummert International, Earth City, MO) and covered by a custom cylindrical plastic cage (30.5 by 4.4 cm). KS4202 is an aphid tolerant soybean variety that has been shown not to impose significant levels of stress on the aphid (Pierson et al. 2010).

Soybean plants used for aphid maintenance and experiments were grown in a greenhouse using 15.2 cm diameter plastic pots and a potting medium comprised of peat moss, perlite, pine bark, and vermiculite (Fafard^®^ 3B Mix, SunGro Horticulture, Agawam, MA). All aphid clonal line maintenance and experiments were carried out in growth chambers at 24 ± 1°C and using a 16:8 h photoperiod.

### Stress treatments and aphid fitness measurements

Soybean plants expressing the *Rag1* gene impose stress on the soybean aphid by reducing survival and reproduction (Hill et al. 2004; Li et al. 2004; Enders et al. 2014). The soybean cultivar Jackson containing the *Rag1* gene (Hill et al. 2006) was used as the plant defensive stress treatment. Technical grade thiamethoxam (Chem Service^®^, Westchester, PA) was used to impose xenobiotic stress. Thiamethoxam is an insecticidal neurotoxin that in the soybean aphid significantly reduced population growth and has an estimated LC_50_ of ~19 ng/mL (Magalhaes et al. 2009). For a nonstressful control treatment we used soybean variety SD01‐76R, a widely used susceptible variety that does not adversely affect aphid fitness (Chiozza et al. 2010). Preliminary experiments screening ten aphid clonal lines for tolerance to plant defenses and insecticides identified significant variation in virulence to *Rag1*‐soybean. From this initial screening four aphid clonal lines demonstrating divergent responses to the *Rag1* gene were selected for use in the current experiment. Lines 1 and 2 showed approximately threefold higher survival compared to lines 3 and 4 after 48 h exposure to *Rag1* soybean (preliminary data not shown). Lines 1 and 2 are therefore considered *Rag1*‐virulent and lines 3 and 4 *Rag1*‐avirulent. Preliminary experiments did not indicate tolerance to thiamethoxam varied across the four clonal lines selected.

To minimize differences in recent rearing conditions, large caged populations of each aphid clonal line were maintained for 2 weeks (~ 2 generations) under standardized conditions in a large walk‐in growth chamber (25°C,16L:8D) prior to collecting adults to generate a large number of same age offspring for use in the experiment. Experimental aphids from each clonal line were age synchronized by setting up eight custom fitted Petri‐dish cages containing 20 adults (collected from the large colony population) placed over a single trifoliate leaf of a nonstressful control plant (SD01‐76R) and allowing reproduction for 24 h. After 24 h all adults were removed and age‐synchronized offspring developed to reproductive age (7 days) prior to being used in experiments. When experimental aphids reached reproductive age a subset of 20 individuals were pooled from across the eight age‐synchronized cages per clonal line and stored at −80°C. These aphids collected before stress treatment (T_0_) were used to determine baseline differences in endosymbiont densities among clonal lines.

For the plant defensive stress treatment, two plants (1 *Rag1* and 1 SD01‐76R) were planted per pot and grown to the V2 vegetative stage. For the insecticide treatment, 1.89 L plastic Gladware^®^ (Glad Manufacturing Co., Rogers, AR) containers were used to make cages that housed two plastic tubes glued in opposite corners, one filled with 10 mL thiamethoxam solution (10 ng/mL dissolved in distilled water) and the other with 10 mL of distilled water (control). Using methods similar to Magalhaes et al. (2009), V1 trifoliate leaves from V2 vegetative stage SD01‐76R plants were excised and immersed in insecticide or control solution for 24 h prior to infestation with experimental aphids. Overall, this design minimized variation between treatments within a pot (*Rag1*) or container (thiamethoxam) and allowed for paired measurements of aphid performance under control and stress treatments.

Age‐synchronized aphids from each of the four clonal lines were randomly pooled from across the eight cages and transferred with a paintbrush to the V1 trifoliate leaf of either a *Rag1*, thiamethoxam treated, or control SD01‐76R plant (20 adults per trifoliate). Four replicate pots were set up per clonal line, each containing one *Rag1* and one SD01‐76R plant custom fitted with a Petri‐dish cage that had a piece of foam secured around the stem of the V1‐trifoliate to prevent movement of aphids between plants in a single pot. Similarly, four replicate plastic containers were set up per clonal line, each containing one thiamethoxam treated and one control SD01‐76R V1‐trifoliate separated by a mesh divider that prevented aphid movement between trifoliates. Aphid survival and nymph production were measured at 24 h (T_24_) for the thiamethoxam treatment and at 48 h (T_48_) for the *Rag1* treatment. Five individuals were randomly harvested from each leaf of each treatment and stored at −80°C for analysis of endosymbiont densities. We were unable to synchronize both stress treatments to 48 h total exposure due to the number of aphids moving off or falling from thiamethoxam treated leaves, which could contribute to variation in insecticide exposure/ingestion. We therefore only used aphids collected directly from a trifoliate leaf across all treatments for measurement of symbiont abundance.

### Estimating endosymbiont abundance in stressed and nonstressed aphids

DNA was extracted from whole aphids using the DNeasy^®^ Blood and Tissue Kit (Qiagen, Valencia, CA) according to manufacturer protocols. We initially screened each aphid clonal line (groups of 10 individuals) using bacterial specific diagnostic PCR with previously published primers (Appendix S1). In addition to the primary symbiont (*Buchnera*), all aphid lines were doubly infected with *Arsenophonus* and *Wolbahia*. Quantitative PCR was used to estimate endosymbiont abundance relative to the aphid host using the following single copy genes: *Arsenophonus* MN cell division protein (ftsK), *Buchnera* chaperonin (GroEL), *Wolbachia* 16s ribosomal gene (16s rRNA) and *Aphis glycines* elongation factor 1*α* (Ef1*α*). Amplification of single copy gene fragments using qPCR provides an estimate of symbiont gene copy number, however due to some symbionts being polypoid (e.g., *Buchnera*) this method estimates genome abundance but not exact number of bacterial cells (Martinez et al. 2014). Target genes for each organism, primer sequences and qPCR efficiency are described in Appendix S1. All qPCR reactions were performed in 10 *μ*L volumes on the BIO‐RAD (Hercules, CA) CFX Connect™ Real‐Time System using iTaq™ Universal SYBR^®^ Green Supermix, with 500 nmol/L of each primer and 5–10 ng input DNA. The following PCR cycling conditions were used for all primer pairs: 95°C for 5 min; 40 cycles of 95°C for 20 sec; 56°C for 20 sec; and 72°C for 30 sec; followed by a 0.5°C increment melt curve from 65 to 95°C. All primers produced a single melt peak. Sanger sequencing of amplicons confirmed target specificity and sequence identity for aphid and symbiont genes. Individual samples were run in triplicate and inter‐run calibrators (Hellemans et al. 2007) using a standard DNA sample were included on each plate to allow for correction of inter‐plate variation as well as a negative control with no DNA template. The negative controls did not show amplification for any of the genes tested from symbionts or aphid host. Endosymbiont abundance was estimated from 7 to 8 individuals per aphid line at T_0_ and 5–12 individuals per aphid line in each treatment group (*Rag1*, thiamethoxam, and control plants) at T_24_ or T_48_.

Endosymbiont densities were calibrated to reflect differences in individual extraction efficiency using the aphid‐host gene Ef1*α*. Samples were calibrated by multiplying each sample C_q_ by a correction factor (CF = maximum Ef1*α* C_q_/sample Ef1*α* C_q_), similar to Martinez et al. (2014). The relative endosymbiont abundance (RA) was estimated as 2^−ΔCq^; where ΔCq = C_q_ (endosymbiont gene)−C_q_ (Ef1*α*). Baseline differences in symbiont abundance (RA) at T_0_ were compared across the four clonal lines using a single‐factor analysis of variance (ANOVA). RA of each endosymbiont was then compared post‐treatment (T_24_ or T_48_) using ANOVA with the following fixed effects model: TREATMENT (*Rag1* or Thiamethoxam vs. control), APHID LINE (1,2,3,4) and the interaction TREATMENT × APHID LINE. In cases where deviations from normality occurred data were log transformed. Post hoc multiple comparisons across aphid clonal lines were performed using Tukey HSD tests on least squared means and *P* values were adjusted for multiple testing. All analyses were implemented in the R statistical environment (R Development Core Team 2012).

### Analysis of host and symbiont stress levels

Stress intensity or stress level can be quantified by measuring the relative change in fitness under stressful and benign conditions [i.e., 1−(Stress/Benign)], such that zero would be no stress and a score of 1 would be the maximum amount of stress (Fox and Reed 2011; Enders et al. 2014). We propose this methodology can be extended to symbiont abundance, whereby reduced titers (i.e., RA) are indicative of adverse effects of stress on bacterial population growth rate within the host. Stress levels were quantified using the following equations for the aphid host (1) and endosymbionts (2):

(1) Aphid Stress Level = 1−(fitness_*Stress*_/fitness_*CON*_)

(2) Symbiont Stress Level = 1−(RA_*Stress*_/RA_*CON*_)

Host stress level was calculated for survival, reproduction and a composite measure (survival × nymph production) from aphids on *Rag1* (fitness_*RAG1*_), thiamethoxam treated (fitness_*Thiam*_), and control (fitness_*CON*_) plants. Survival was measured as the number of adults alive at T_24_ or T_48_ and nymph production was measured as the average number of offspring produced per individual female alive. RA was used to measure endosymbiont stress levels.

The experimental design, whereby each replicate pot or plastic container had paired stress and control treatments, enabled stress levels to be estimated for each replicate pot (Rag1 stress) or container (thiamethoxam stress) separately (four replicates/ aphid line/ stress treatment). First, aphid stress levels for each fitness measure (survival, reproduction, cumulative fitness) were compared across all four clonal lines using a single‐factor ANOVA for plant defensive and xenobiotic stress separately. Second, we exploited phenotypic variation in clonal virulence to *Rag1* to investigate the relationship between aphid and symbiont responses [i.e., stress levels equations (1) and (2)] to plant defensive stress and determine whether this relationship varied across the clonal lines. We performed an ANCOVA on aphid stress level calculated for cumulative fitness (APHID S_LEVEL) with the following variables: covariate SYMBIONT S_LEVEL, APHID LINE, and the interaction APHID LINE × SYMBIONT S_LEVEL. Separate analyses were run for each endosymbiont, implemented in the R statistical environment (R Development Core Team 2012).

### Aphid clonal line genotyping and Arsenophonus MLST

A multi‐locus approach with six microsatellite loci was used to genetically distinguish the four aphid clonal lineages (Table 1). DNA extracted from six randomly chosen individuals per aphid clonal line used to estimate endosymbiont abundance was also used to genotype the aphid host. Amplification of microsatellite loci was performed in 20 *μ*L reaction volumes using an Amresco^®^ (Solon, OH) PCR Kit with fluorescently labeled forward primers, 500 nmol/L concentration of both forward and reverse primers, and 5–10 ng of DNA. Cycling conditions were as follows: 95°C for 5 min; 30 cycles of 95°C for 30 sec; 55°C for 30 sec; and 72°C for 45 sec; with final extension of 70°C for 10 min. Samples were genotyped using an Applied Biosystems^®^ (Thermo Fisher Scientific Waltham, MA) 3130 instrument and allele sizes determined using GeneMapper^®^ software (Applied Biosystems, Thermo Fisher Scientific, Watlham, MA).

**Table 1:** Genotypes of four aphid clonal lines at six microsatellite loci

Aphid line	Allele sizes (bp) at each diploid locus											
	Ago66^a^		Ago89^a^		AF48^a^		Agl1‐2^b^		Agl1‐10^b^		Agl1‐22^b^	
	Allele 1	Allele 2	Allele 1	Allele 2	Allele 1	Allele 2	Allele 1	Allele 2	Allele 1	Allele 2	Allele 1	Allele 2
1	152	156	151	153	301	301	232	244	219	233	190	190
2	152	156	151	153	301	301	232	244	219	233	190	190
3	150	152	151	151	301	301	232	244	219	233	190	190
4	150	152	151	153	301	301	244	244	219	219	190	190

a Kim et al. (2010).

b Michel et al. (2009).

We employed a multi‐locus sequence type (MLST) approach to investigate *Arsenophonus* genetic diversity using four genes (*fbaA, ftsK, spoT,* and *yaeT)* and the 23s‐16s rRNA intervening region. Wulff et al. (2013) previously used *fbaA, ftsK, yaeT*, and the 23s‐16s rRNA for MLST analysis of *Arsenophonus* from soybean aphids. Additionally, we included the *spoT* gene using primers published by Jousselin et al. (2013). DNA was amplified from two experimental individuals per aphid line using an Amresco^®^ PCR Kit with 500 nmol/L each primer and 5–10 ng of DNA per reaction. PCR cycling conditions were as follows for all five genes: 95°C for 5 min; 30 cycles of 95°C for 30 sec; 56°C for 90 sec; and 72°C for 30 sec; with final extension of 60°C for 15 min. PCR products were cleaned up using the Affymetrix USB^®^ (Thermo Fisher Scientific, Waltham, MA) ExoSAP‐IT kit according to manufacturer protocols and sequencing was performed at the University of Nebraska Medical Center DNA Sequencing Core Facility. MLST primer sequences and amplicon lengths are reported in Appendix S1. Sequences for each gene were submitted to Genbank.

## Results

### Effect of stress on aphid fitness

**Figure 1:**
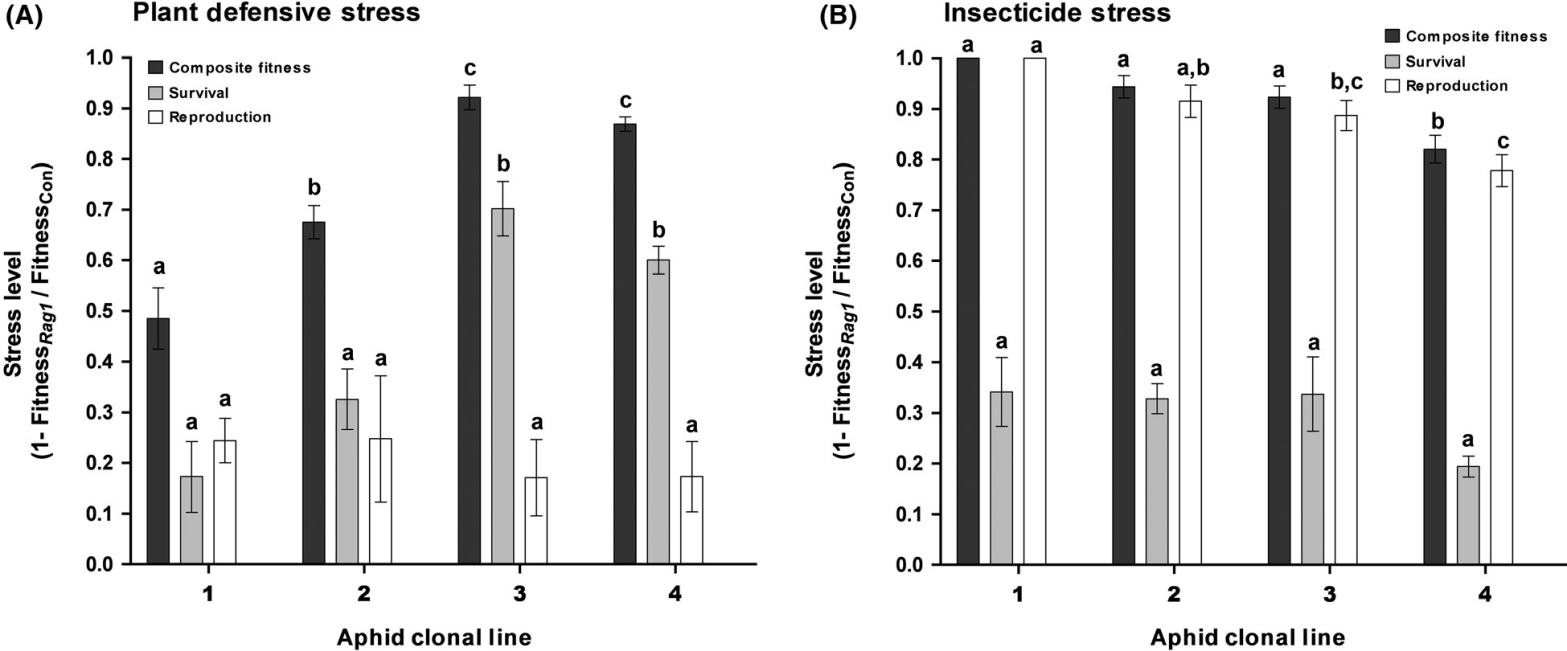
Aphid stress levels induced by exposure to (A) an aphid‐resistant soybean variety for 48 h or (B) soybean treated with the insecticide thiamethoxam for 24 h. Stress level (±SE) is measured as the reduction in fitness under stress(*Rag1* or thiamethoxam treated leaves) compared to nonstressful control plants (SD01‐76R). Composite fitness was calculated as Survival × Reproduction. Letters indicate significant differences in post hoc pairwise comparisons between aphid clonal lines (1–4) within each fitness measure (*P *<* *0.05).

Exposure to *Rag1* soybean plants significantly reduced aphid survival and reproduction relative to control plants and aphid clonal lines showed significant variation in response to plant defensive stress (Fig. 1A). Aphid mortality was low on control plants across all aphid clonal lines after 48 h (6 ± 1%), but mortality ranged from 31 ± 4% (virulent lines 1 and 2) to 67 ± 3% (avirulent lines 3 and 4) on *Rag1* plants. In terms of stress level, adult survival was reduced by 65 ± 3% in avirulent lines compared to only 25 ± 5% in virulent lines. Nymph production was equivalently reduced in aphids on *Rag1* plants by 21 ± 4% across all lines, resulting in similar reproductive stress levels (Fig. 1). On average stressed aphids produced 2.5 ± 0.13 nymphs per female in 48 h compared to an average 3.3 ± 0.17 nymphs produced by unstressed aphids. When the effect of plant defensive stress was combined for both fitness components, reductions in composite fitness showed significant variation across aphid clonal lines (Fig. 1). Clonal lines 3 and 4 suffered greater reductions in overall fitness when exposed to *Rag1* soybean compared to lines 1 and 2. In particular, virulent clonal line 1 performed significantly better (i.e., exhibited lowest stress levels) on *Rag1* plants compared to all other aphid lines. Overall, our results show significant quantitative variation in *Rag1*‐virulence across the four clonal lines based on survival and composite fitness measures.

Xenobiotic stress significantly reduced all fitness components, with similar effects across clonal lines (Fig. 1B). Mortality on control leaves was low (5 ± 1%), but 33 ± 3% of aphids died within 24 h of exposure to leaves treated with the insecticide thiamethoxam. Reproduction was also adversely effected, stressed females produced on average less than one nymph, with clonal line 3 producing no offspring. In contrast, unstressed females produced 1.9 ± 0.1 nymphs on average in 24 h. The overall effect of thiamethoxam stress was to reduce composite fitness by more than 90% in 3 of 4 clonal lines and 82 ± 3% in the remaining line, indicating there was minor variation in tolerance to the insecticide across aphid clonal lines.

### Effect stress on endosymbiont relative abundance

**Figure 2:**
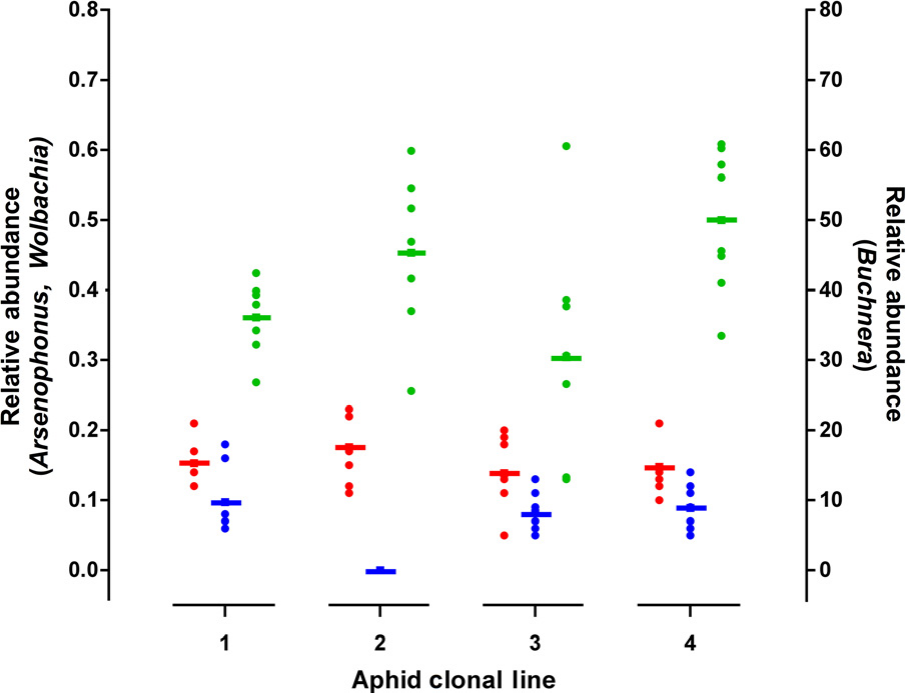
Relative abundance measured using qPCR of the obligate endosymbiont *Buchnera* (green) and two facultative endosymbionts *Arsenophonus* (red) and *Wolbachia* (blue) in soybean aphids before treatment on nonstressful control plants (SD01‐76R). Solid bars represent the average relative abundance for each aphid clonal line (1–4), with circles representing individual aphids.

We first examined whether the four aphid clonal lines exhibited baseline differences in endosymbiont titer prior to exposure to stressful host‐plant defenses or insecticide (T_0_). The relative abundance of *Arsenophonus* was equivalent across clonal lines, while there were minor differences in *Buchnera* and *Wolbachia* titers (Fig. 2, Table 2A). Overall, aphid lines 2 and 3 differed in *Buchnera* relative abundance and line 2 had significantly lower *Wolbachia* densities compared to all other lines.

**Table 2:** Analysis of variance comparing relative abundances of endosymbionts across four soybean aphid clonal lines (1–4) prior to stress treatment (A) and when exposed to plant defensive, insecticide stress or control conditions (Treatment: *Rag1* vs. SD01‐76R plant) for 48 h (B,C). Tukey HSD post hoc pairwise comparisons were performed between aphid clonal lines

Source	DF	*Buchnera*	*Arsenophonus*	*Wolbachia*
		*F* value	*F* value	*F* value
(A) Pretreatment (T_0_)				
Aphid line	3	9.48***	6.00***	48.30***
Line comparisons				
1 vs. 2		NS	NS	***
1 vs. 3		NS	NS	NS
1 vs. 4		NS	NS	NS
2 vs. 3		NS	NS	***
2 vs. 4		NS	NS	***
3 vs. 4		*	NS	NS
Residuals	26			
(B) Post‐treatment: plant defensive stress				
Treatment	1	22.00***	7.92**	6.31*
Aphid line	3	9.48***	6.00***	48.30***
Line comparisons				
1 vs. 2		NS	**	***
1 vs. 3		***	*	***
1 vs. 4		NS	**	NS
2 vs. 3		*	NS	***
2 vs. 4		NS	NS	***
3 vs. 4		***	NS	***
Treatment × Aphid line	3	0.08	0.01	0.996
Residuals	92			
(C) Post‐treatment: insecticide stress				
Treatment	1	13.68***	4.61*	5.15*
Aphid line	3	4.46*	4.70**	26.91***
Line comparisons				
1 vs. 2		NS	NS	***
1 vs. 3		NS	NS	NS
1 vs. 4		NS	NS	NS
2 vs. 3		*	**	**
2 vs. 4		NS	NS	***
3 vs. 4		NS	NS	***
Treatment × Aphid line	3	0.27	2.02	1.2
Residuals	32			

*P *< ***0.001 **0.01 *0.05 NS > 0.05.

**Figure 3:**
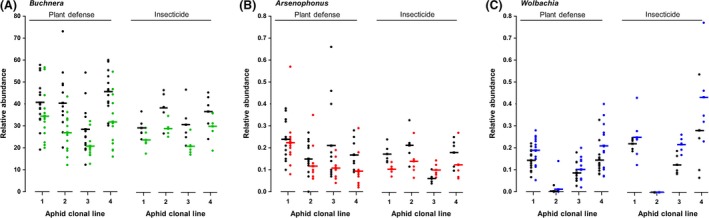
Relative abundance measured using qPCR of the endosymbionts *Buchnera* (A), *Arsenophonus* (B) and *Wolbachia* (C) in soybean aphids after exposure to nonstressful control plants (SD01‐76R: black circles), plant defensive stress (*Rag1*) or insecticide stress (Thiamethoxam). Solid bars represent the average relative abundance for each aphid clonal line (1–4), with circles representing individual aphids. Note the experimental design paired each replicate stress treatment with a control treatment.

When aphids were exposed to host‐plant mediated stress endosymbiont abundances were significantly altered and unique changes were observed for obligate and facultative symbionts (Fig. 3A–C). The relative abundance of the primary endosymbiont *Buchnera* was approximately 100‐fold higher than both facultative symbionts (*Arsenophonus* and *Wolbachia*), which showed similar titer levels. When aphids were exposed to stressful *Rag1* plants *Buchnera* relative abundance decreased on average by 26 ± 4% compared to aphids fed on nonstressful control plants (Fig. 3A, Table 2B). All aphid lines showed equivalent reductions in *Buchnera* abundance (Treatment × Aphid Line: *P *>* *0.05), but post hoc multiple comparisons indicated that aphid line 3 had a significantly lower overall *Buchnera* titer compared to the other clonal lines (Table 2B). *Arsenophonus* relative abundance was significantly reduced by 30 ± 9% in aphids exposed to soybean defenses associated with the *Rag1* gene (Fig. 3B, Table 2B). Similar to the primary symbiont, the effect of exposure to *Rag1* soybean on A*rsenophonus* abundance did not vary across the four aphid lines (Table 2B, Fig. 3B). However, aphid line 1 had an overall higher *Arsenophonus* titer compared to all other lines (Fig. 3B). In contrast to the reductions observed in *Buchnera* and *Arsenophonus*, the relative abundance of *Wolbachia* significantly increased on average by twofold in stressed aphids compared to unstressed aphids (Fig. 3C). *Wolbachia* titer levels also varied significantly across the aphid lines (Table 2B), an effect primarily driven by the extremely low levels of *Wolbachia* found in aphid line 2 (Table 2B, Fig. 3C).

Exposure to xenobiotic stress produced a similar overall pattern of changes to symbiont relative abundance (Fig. 3A–C, Table 2C). Aphids fed on leaves treated with thiamethoxam had on average 23 ± 3% lower *Buchnera* titers compared to unstressed aphids, an effect that was similar across clonal lines (Treatment × Aphid Line: *P *>* *0.05). Post hoc multiple comparisons indicated that line 3 had a lower overall *Buchnera* titer than line 2 (Table 2C). *Arsenophonus* abundance decreased on average by 12 ± 22% in stressed aphids, with only clonal lines 2 and 3 differing in overall titer (Table 2C). Although aphid line 4 showed increased *Arsenophonus* titer in stressed aphids (see Fig. 3B), there was no overall difference in the effect of stress across clonal lines (Treatment × Aphid Line: *P *>* *0.05). As with plant defensive stress, *Wolbachia* significantly increased 1.5‐fold in insecticide stressed aphids. The low *Wolbachia* titer found in aphid line 2 was again primarily responsible for observed aphid clonal variation (Table 2C).

### Relationship between aphid virulence and symbiont stress levels

**Figure 4:**
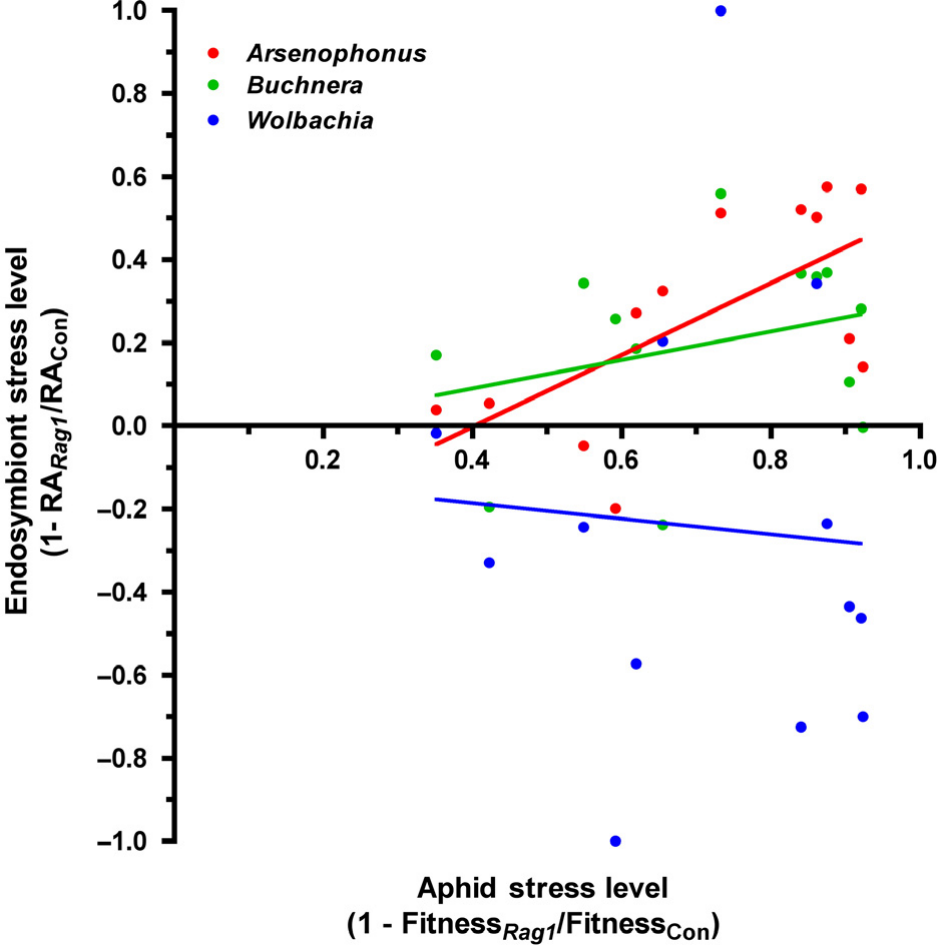
Relationship between aphid host and endosymbiont stress levels when exposed to plant defenses (*Rag1* gene). Aphid‐host stress levels were calculated using composite fitness (survival × reproduction) and symbiont stress levels were calculated using relative abundance (RA). Lines represent linear regression of host and symbiont stress levels.

We performed an ANCOVA of host and symbiont stress levels to investigate the relationship between response of the aphid and its endosymbionts to stress inflicted by soybean plant defenses and determine whether there was variation across aphid clonal lines. Stress levels were calculated from pairs of *Rag1* and control plants grown together in single pot (see Methods), which differed from the previously described analysis of aphid fitness and symbiont abundance that averaged across individuals from all pots. Stress levels when exposed to plant defenses (*Rag1*) were positively correlated between the aphid host and the facultative symbiont *Arsenophonus* (*r* = 0.65, *t* = 2.87, *P *=* *0.02), however there was no significant relationship for *Buchnera* or *Wolbachia* (Fig. 4). The slope of the relationship between soybean aphid and *Arsenophonus* stress levels was 0.45 ± 0.17 (*F*_1,5_ = 26.59, *P *<* *0.001), which predicts *Arsenophonus* abundance is less affected in aphids with higher relative fitness on *Rag1* plants. We observed substantial variation in *Buchnera* and *Wolbachia* stress levels, which were uncorrelated with aphid stress levels (slopes = 0.23 ± 0.24 and −0.03 ± 0.14 respectively, *P* values >0.05). Overall, these relationships between aphid and endosymbiont stress levels did not vary depending on aphid clonal line (Aphid Line × Symbiont Stress Level, *P* values >0.05). Consistent with previous ANOVA (see Fig. 1), cumulative stress levels varied significantly across the aphid lines in all three analyses corresponding to each endosymbiont (*P* values <0.05).

### Aphid genotyping and Arsenophonus MLST strain diversity

Three unique multi‐locus genotypes were found among the four aphid clonal lines using six microsatellite markers (Table 1). Virulent aphid lines 1 and 2 were identical at all loci examined, but produced phenotypically distinct responses to *Rag1* defenses (Table 2). Avirulent lines 3 and 4 were genetically distinct from one another, but showed equivalent reductions in fitness when exposed to *Rag1* (Table 2).

We investigated whether aphid lines harbored unique strains of *Arsenophonus* by measuring genetic diversity using an MLST approach with five genes (Appendix S1). All sequences generated across the four aphid lines were identical for the five *Arsenophonus* genes examined.

## Discussion

In this study, we investigated changes in soybean aphid‐microbial dynamics resulting from exposure to biotic stress imposed via host‐plant defenses and abiotic stress associated with insecticide exposure. Our findings reveal reduced soybean aphid fitness under host‐plant defensive and xenobiotic stress is accompanied by variable changes in obligate and facultative symbiont abundances (Figs. 1, 3). In addition, increased aphid clonal virulence to the *Rag1* defense gene was positively correlated with decreased *Arsenophonus* stress levels (Fig. 4). Taken together our results demonstrate divergent responses between obligate and facultative symbionts to stress inflicted on the host aphid. However, similar overall patterns of altered endosymbiont abundance were found in response to plant defenses and xenobiotic challenge, suggesting general stress‐induced responses within the microbiome exist as well.

### Altered endosymbiont abundances under stress

Recent transcriptomic and proteomic studies indicate symbionts are highly responsive to the internal environment of the aphid host (Nguyen et al. 2009; Francis et al. 2010; Enders et al. 2015). When challenged by environmental stress, coregulation of molecular responses can occur, such as the simultaneous up‐regulation of aphid and *Buchnera* molecular chaperones (Enders et al. 2015). However, depending on the nature of the relationship with the host, aphid symbionts may exhibit varying responses to stress‐induced changes in host physiology. In the soybean aphid, exposure to two different stressors caused symbiont specific responses rather than a general pattern of community wide reduced abundance. Previous work in the white fly *Bemisia tabaci* also found that facultative and obligate symbiont densities are differentially affected by host plant and tolerance to insecticides (Ghanim and Kontsedalov 2009; Pan et al. 2013). However, in the current study obligate and facultative symbionts responded similarly to different stress types (Fig. 3A–C). Recent transcriptional profiling of *Rag1*‐stressed soybean aphids identified xenobiotic challenge, potentially associated with toxic plant secondary metabolites, as a primary cause of stressful aphid‐*Rag1* interactions (Bansal et al. 2014b). Exposure to insecticides and chemical defenses associated with *Rag1* may therefore have similar physiological effects on the aphid host, which could in part explain the common stress‐induced changes to the microbiome observed in this study.

In this study, *Buchnera* showed a consistent pattern of stress that was independent of aphid clonal differences in *Rag1*‐virulence or tolerance to insecticide. This result suggests the metabolic interdependence of obligate symbiont and insect host may increase sensitivity to external stress, such that a low threshold level of aphid stress will adversely affect abundance. Similar reductions in *Buchnera* density have been observed in soybean aphids fed on virus‐infected plants (Cassone et al. 2015) and in the pea aphid following heat shock (Dunbar et al. 2007). Age and aphid genotype have also proven to be primary factors contributing to changes in *Buchnera* titer in *A. pisum* (Vogel and Moran 2011; Martinez et al. 2014). However, in *A. glycines* we found only minor variation in overall *Buchnera* titer level among clonal lines (Table 2). Our results and previous studies suggest changes in *Buchnera* density may vary depending on stress type and specific changes in host internal environment. For example, stressors causing irreversible damage within the host may consistently reduce *Buchnera* populations. Further work is needed to investigate whether alternative forms of stress, including additional *Rag*‐mediated defenses, cause similar effects on the obligate symbiont of the soybean aphid.

Facultative symbionts exhibited contrasting changes in abundance in stressed and unstressed soybean aphids (Fig. 3B,C). Impaired physiological function and cellular damage that create a suboptimal aphid internal environment for the symbiont or disrupt normal host‐symbiont interactions, could generally contribute to reduced *Arsenophonus* density. Increases in *Wolbachia* abundance could however result from compromised host immunity under stress. Research documenting higher densities of *Wolbachia* in mosquitos (*Culex pipiens*) carrying insecticide resistance genes, indicates the physiological cost to resistance may impair host immune function and thus control over microbial populations (Berticat et al. 2002; Echaubard et al. 2010). Likewise, host‐plant mediated and xenobiotic stress may interfere with normal aphid immune function, thus allowing pathogenic or parasitic symbionts to multiply. Although *Wolbachia* is known to influence reproductive processes in a number of arthropods (Werren et al. 2008), it is unclear what effect, if any, this symbiont may have on *A. glycines*. *Wolbachia* was undetectable in several individuals of clonal line 2, but offspring production was equivalent among clonal lines under stress. In general, our results highlight a need for further research investigating the impact of stress on facultative symbionts within aphids, and in particular the molecular underpinnings of symbiont‐aphid interactions.

### Do symbionts contribute to variation in soybean aphid stress tolerance?

In this study, facultative symbionts of the soybean aphid do not appear to enhance tolerance to xenobiotic stress, although further work using a variety of toxins is needed to confirm the generality of our findings. We were however able to exploit natural variation in *A. glycines* virulence against soybean defenses to examine the extent to which changes in endosymbiont densities are associated with the outcome of aphid‐host plant interactions. Specifically, soybean aphid virulence to *Rag1‐*mediated defense was found to correlate with stress‐induced changes in *Arsenophonus* abundance, but not *Buchnera* or *Wolbachia* (Fig. 4).

What factors could be responsible for generating the observed association between aphid *Rag1*‐virulence and changes in *Arsenophonus* abundance? One hypothesis is *Arsenophonus* is mediating interactions with the host plant, for example, through mechanisms that increase tolerance to or interfere with soybean defenses. Recent studies in the western corn rootworm (*Diabrotica virgifera virgifera*) and the tomato psyllid (*Bactericera cockerelli*) have found higher concentrations of endosymbionts correlate with reduced expression of plant defensive pathways (Barr et al. 2010; Casteel et al. 2012). In addition, gut symbionts of several insects produce digestive enzymes that counteract and degrade plant defensive compounds (Sugio et al. 2014). Higher densities of beneficial symbionts could therefore increase production of detoxification enzymes or effector proteins that interfere with plant defensive signaling. However, recent work using methods to experimentally cure aphids of *Arsenophonus* found that soybean aphid virulence was not dependent on infection status, although infected populations did generally perform better (Wulff and White 2015). An alternative hypothesis is that changes in *Arsenophonus* titer observed in the current study are a by‐product of stress‐induced physiological changes that correlate with reduced fitness and therefore aphid virulence (i.e., *Rag1*‐virulent aphids are less stressed and so are their *Arsenophonus* populations). Additional research is needed to investigate the underlying mechanisms that contribute to general *Arsenophonus* derived fitness benefits observed by Wulff and White (2015), but also whether molecular interactions with the aphid host vary depending on stress intensity.

Finally, we investigated whether *Arsenophonus* strain diversity contributed to differences in the virulence of aphid clones to *Rag1* soybean. Consistent with Wulff et al. (2013) we found one *Arsenophonus* MLST genotype across all aphid clonal lines, suggesting strain differences are not primarily responsible for variation in *Rag1‐*virulence. Furthermore, Wulff and White (2015) found that artificially infecting *Rag1*‐avirulent aphids with *Arsenophonus* isolated from *Rag1*‐virulent aphids did not improve performance on *Rag1* plants. Aphid clonal differences, rather than *Arsenophonus* mediated benefits alone, appear to be driving variation in aphid response to plant induced stress. Changes in relative abundance observed in this study could result from host specific interactions, whereby *Arsenophonus* is responding to internal environment changes unique to each aphid clonal lineage or genotype. This differs from the response of *Buchnera*, which generally appeared more sensitive to stress and was adversely affected at lower aphid stress levels.

### Conclusions and future directions

Symbiont‐driven adaptive traits are often viewed as one sided, where the microbe is primarily responsible for observed phenotypic variation in the host. However, a growing body of work suggests dynamic interplay between the host and its symbiont community determines holobiont response to environmental challenge (Rosenberg et al. 2009; Gilbert et al. 2010; Martinez et al. 2014). Multi‐trophic approaches that examine aphid symbioses in an ecological context are needed to complement studies focused on the isolated effects of specific symbionts. This will facilitate an improved understanding of host versus symbiont contributions to insect stress response and shed light on interactions within the microbiome that affect adaptive processes, in particular, under what circumstances symbionts provide conditional benefits to their hosts and how symbiont‐driven evolution proceeds within the context of multi‐stress environments.

As the evolutionary processes that contribute to complex trophic networks continue to unfold, we are beginning to understand how the insect microbiome responds to the host environment and how symbiont players interact. This study adds to growing evidence that environmental stress causes distinct changes in insect endosymbiont communities, which in the long‐term could lead to adaptive differences in within‐host ecology. While the acute effects of stress on insect symbiont dynamics have received attention, it remains unclear just how rapidly endosymbionts respond to stress imposed on the host and whether changes are transient or sustained over time. Research investigating whether prolonged or repeated bouts of stress alter aphid microbiome diversity and structure could improve our understanding of the ecological and adaptive significance of insect‐microbial associations.

## Data Accessibility

Fitness and qPCR data will be archived in Dryad and MLST sequence data will be submitted to GenBank.

## Conflict of Interest

None declared.

## Supporting information


**Appendix S1.** Target gene primer information for symbionts and aphid host used in qPCR and genes used for MLST approach.
