# Supplementary material for: Stress‐induced changes in abundance differ among obligate and facultative endosymbionts of the soybean aphid
Source: Ecol Evol. 2016 Jan 18;6(3):818–29. doi: 10.1002/ece3.1908 (PMC4739556; doi:10.1002/ece3.1908)
Supplement: Supplementary file 1 — Appendix S1. Target gene primer information for symbionts and aphid host used in qPCR and genes used for MLST approach. [file ECE3-6-818-s001.docx]

**Supporting Information**

**Appendix S1:** Target gene primer information for symbionts and aphid host used in qPCR and genes used for MLST approach. qPCR primer efficiency and R^2^ were determined from linear regression of a standard curve based on a serial dilution of gDNA ranging from 100ng-0.1ng.

| **Organism** | **Gene Name** | **Primer** | **Primer Sequence (5’-3’)** | **Genebank Sequence** | **Amplicon (bp)** | **Efficiency (%)** | **R^2^** |
| --- | --- | --- | --- | --- | --- | --- | --- |
| **Diagnostic Primers** |  |  |  |  |  |  |  |
| *Arsenophonus* | Ars23S-1^1^ | For | CGTTTGATGATTCATAGTCAAA |  |  | NA | NA |
|  | Ars23S-2^1^ | Rev | GGTCCTCCAGTTAGTGTTACCCAAC |  |  |  |  |
| *Buchnera* | 757F^2^ | For | GAGGAATACCYKTGGCGAAA |  |  | NA | NA |
|  | 1507R^2^ | Rev | TACCTTGTTACGACTTCACCCCAG |  |  |  |  |
| *Wolbachia* | wRi23s-F^3^ | For | GGCGTACCTTTTATCCGTTG |  |  | NA | NA |
|  | wRi23s-R^3^ | Rev | GAGATTCTGTTAGTAGTGACGAG |  |  |  |  |
| **qPCR Primers** |  |  |  |  |  |  |  |
| *Aphis glycines* | EF1α | For | CGCACCTGGTCACAGAGATT | EU358911.1 | 135 | 101.3 | 0.99 |
|  |  | Rev | TGCTCACGGGTTTGTCCATT |  |  |  |  |
| *Arsenophonus* | ftsK | For | TCAAGGTGGCGCTGAATCTT | KC701198.1 | 121 | 101.8 | 0.99 |
|  |  | Rev | CGGGCTTACCTCTAGCTTTCC |  |  |  |  |
| *Buchnera* | GroEL | For | GCTGCTTCTGTTGCTGGTTT | KJ543522.1 | 116 | 98.0 | 0.99 |
|  |  | Rev | CCCATACCACCCATTCCACC |  |  |  |  |
| *Wolbachia* | 16s rRNA | For | TGGAGGAAGGTGGGGATGAT | JN109116.1 | 112 | 100.6 | 0.96 |
|  |  | Rev | AGGATTAGCTCAGCCTTGCG |  |  |  |  |
| **MLST Primers** |  |  |  |  |  |  |  |
| *Arsenophonus* | fbaA | For | ATGTGCATCAAATGGCAAAA | KC701199.1 | 499 | NA | NA |
|  |  | Rev | TTTTCCGCTGGCAAATTAAA |  |  |  |  |
| *Arsenophonus* | ftsK | For | CAGCCGGTATTCACCTTGTT | KC701198.1 | 334 | NA | NA |
|  |  | Rev | CCATTACCACTCTCACCCTCA |  |  |  |  |
| *Arsenophonus* | spoT^4^ | For | GGAGAATCTAGCACAACHGCTC |  | 703 | NA | NA |
|  |  | Rev | GGGCGACAACATTTRGCAAAGG |  |  |  |  |
| *Arsenophonus* | yaeT | For | ATATGCCTGTTCGGGTAGGA | KC701197.1 | 345 | NA | NA |
|  |  | Rev | GCGGTAGCGGTGTGACTACT |  |  |  |  |
| *Arsenophonus* | 23s-16s rRNA^1^ | For | CGTTTGATGATTCATAGTCAAA | KC019882 | 581 | NA | NA |
|  |  | Rev | GGTCCTCCAGTTAGTGTTACCCAAC |  |  |  |  |
| ^1^Wulff et al 2013, ^2^Russell and Moran 2005, ^3^Lui et al 2012, ^4^Josselin et al 2012 | | | |  |  |  |  |
